# Improving Essential Oil, Pigments, and Mineral Accumulation in Basil Through Integrated Silicon and Nano‐Fertilizer Treatments in Aquaponics

**DOI:** 10.1002/fsn3.71961

**Published:** 2026-06-02

**Authors:** Al‐Hatem JihanYahya, AsmaaMohammed Adil, AliFarouq Al‐Ma'athedi, Heidar Meftahizade

**Affiliations:** ^1^ Department of Horticulture and Landscape, College of Agriculture and Forestry University of Mosul Mosul Iraq; ^2^ Department of Horticultural Science, Faculty of Agriculture and Natural Resources Ardakan University Ardakan Iran

**Keywords:** aquaculture, basil, essential oil, medicinal plants, mineral accumulation, nano‐fertilizer

## Abstract

Innovative agricultural production systems and effective nutrient management approaches are essential to meet an escalating global demand for sustainable food. The influence of three delivery systems: silicon (Si), a nano‐fertilizer (Nanogreen), and a chelated micronutrient fertilizer (Disper Complex GS) was evaluated on *Ocimum basilicum* L. growth (height and fresh weight), physiological characteristics (photosynthetic pigments), the amount of macro and micronutrients absorbed, and essential oil yield in a closed aquaponic growing system. A completely randomized design (CRD) based on a Taguchi L9 orthogonal array was used, and utilized three replications within each treatment. The treatments consisted of three levels of Si (0, 0.1, and 0.2 mM), three levels of the nano‐fertilizer (0, 1, and 2 g L^−1^), and three levels of Disper (0, 2, and 4 g L^−1^). All fertilizers were applied as foliar sprays so as to avoid disruption of the fish's aquaponic water and to maintain fish safety. Overall, each of the treatment combinations produced statistically significant improvements over the controls in plant growth, total biomass production, photosynthetic pigment accumulation, essential oil yields, and macro‐ and micronutrient content. In addition, the maximum improvements in plant height, fresh weight, chlorophyll content, essential oil yield, and macro‐ and micronutrient accumulation occurred when 0.2 mM Si, 2 g L^−1^ of the nano‐fertilizer, and 4 g L^−1^ Disper were applied. However, antioxidant activity decreased with high input treatments, suggesting lower levels of oxidative stress due to a higher level of nutrition. Correlation and regression analysis indicated significant positive correlations between growth, mineral nutrient uptake, and pigment accumulation, with zinc being a critical mineral. These findings provide an effective strategy for sustainable basil production in aquaponic systems through optimized nutrient management.

## Graphical abstract

Combined Si and nano‐fertilizers enhanced basil growth and biomass. Essential oil, pigments, and mineral content significantly increased. Integrated nutrition improved yield and quality in aquaponic systems.

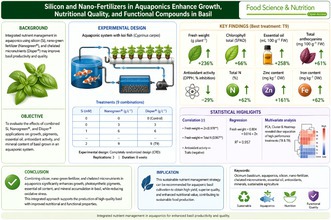

## 1 Introduction

Sweet Basil ( *Ocimum basilicum* L.) is an aromatic vegetable from the Lamiaceae family with worldwide edible and therapeutic uses (Kamelnia et al. 2023; Romano et al. 2022; Ziaei et al. 2014). Although basil is originally native to the Americas, tropical Africa, and Asia, it is now widely cultivated in Europe, North Africa, and south‐west Asia (Bozyel et al. 2024). This plant is used as a vegetable and herb in cooking and food industries due to its aroma and nutritional value (Azizah et al. 2023). Sweet Basil contains phenolic compounds, flavonoids, and volatile oils with antioxidant and antimicrobial properties, which enable it to play a role in reducing chronic inflammation, protecting against oxidative stress, and improving liver and kidney function (Romano et al. 2022). Basil extract displays excellent medicinal possessions, including antiviral, antibacterial, antifungal, and antioxidant activity, antidiabetic potential, neuroprotective qualities, and anticancer properties (Azizah et al. 2023; Kamelnia et al. 2023; Zhakipbekov et al. 2024). The volatile secondary metabolites of basil comprise alkaloids, phenolics, flavonoids, tannins, saponins, cardiac glycosides, and steroids (Al‐Snafi 2021; Benedec et al. 2012; Güez et al. 2017; Kwee and Niemeyer 2011; Zhakipbekov et al. 2024). In addition to its well‐documented medicinal and nutritional properties, basil has attracted increasing attention in controlled‐environment agriculture systems such as aquaponics, where plant growth, phytochemical accumulation, and resource‐use efficiency can be simultaneously optimized.

Outdoor agriculture faces many challenges, including soil‐borne diseases, lack of precise control of nutrients at different stages of plant growth in the soil, difficulty of weed control, low efficiency of water consumption, and impossibility of controlling environmental conditions. Indoor farming technologies such as hydroponics and aquaponics are crucial for advancing sustainable agriculture, especially in urban areas with limited land for conventional agriculture (Heo et al. 2024). Therefore, in recent years, professional gardeners have shown interest in producing high‐quality horticultural crops under controlled conditions such as hydroponic and aquaponics cultivations (Derikvand et al. 2024). In these cultivation systems, plant diseases and water and nutrients are precisely controlled (Kotzen et al. 2019; Rajaseger et al. 2023; Tetra et al. 2023), thus, the photosynthesis rate and plant yield were significantly higher than in the soil‐based system (El‐Shal et al. 2022; Majid et al. 2021). In the *Acmella oleracea* (L.) the content of total phenolics, flavonoids, and antioxidants in plants cultivated under hydroponic condition was higher than in field and tissue culture (Abeysinghe et al. 2014). Aquaponic systems can enhance plant and fish productivity, reduce land use, and improve sustainability (Mehdi et al. 2026).

Aquaponics is an agricultural method that leverages the symbiotic relationship between fish and plants in a unique combination of recirculating aquaculture system (RAS) and hydroponics in a closed‐loop system (Goddek et al. 2015). Recently, due to the decline of freshwater resources, aquaponics systems have emerged as a sustainable solution in integrated agriculture‐aquaculture systems (IAAS) based on water recycling and optimization (Dodangodage et al. 2026; Goda et al. 2025; Verma et al. 2023). This system relies on a combination of plant cultivation and aquaculture, in which nitrifying bacteria such as Nitrosomonas and Nitrobacter convert nitrogenous fish waste into nitrates that can be absorbed by plants (Adhikari et al. 2020; Al Tawaha et al. 2025; Thakur et al. 2023). In this system, nutrient film technique (NFT) pumps nutrient solution through water channels, which progresses nutrient absorption efficiency and supports optimal growth of plants (Hussain and Brown 2025). To ensure nutritional balance in aquaponic systems, nutrient deficiencies are addressed by spraying the vegetation with environmentally safe fertilizers to compensate for elements such as phosphorus, potassium, iron, zinc, and others, which may not be sufficiently provided by fish waste (Hutagalung et al. 2023). It is necessary to adjust their concentrations to maintain the health of aquatic organisms and the stability of the ecosystem (Hutagalung et al. 2023; Lobanov et al. 2025; Rajaseger et al. 2023; Stoyanova et al. 2024; Zaili et al. 2024).

Nano‐fertilizers are an advanced choice for improving the efficiency of nutrient absorption and reducing losses (Demeke et al. 2025; Elbanna et al. 2024; Kekeli et al. 2025). They have proven effective in increasing chlorophyll concentrations and medicinal compounds, and in increasing antioxidant activity, compared to conventional fertilizers (Elbanna et al. 2024; Rodgers et al. 2022). Recent studies demonstrate that adding complementary fertilizers to aquaponic systems improved basil plant growth and chlorophyll index, compared to conventional agricultural systems (Mourantian et al. 2023; Rodgers et al. 2022).

Although aquaponic systems depend mainly on fish‐based nutrients to grow crops, several studies show that aquaponics has limited amounts of some important nutrients such as iron and potassium, which plants need to grow properly. Because of the way aquaponic systems are designed, iron and potassium can often be deficient because of precipitation and not being very soluble in recirculating systems. Therefore, to remediate the nutrient deficiency in aquaponic systems, targeted supplemental feeding programs (particularly those that utilize foliar feeding) have been proposed as a means of providing the needed nutrients, while not adversely affecting the fish health or the performance of the biofilter.

In this study, we hypothesized that the combined application of silicon, nano‐fertilizer (Nanogreen), and chelated micronutrient fertilizer (Disper Complex GS) in a closed NFT aquaponic system would synergistically enhance basil growth while simultaneously improving its nutritional quality and bioactive properties. Specifically, it was assumed that this integrated fertilization strategy would increase photosynthetic pigments, mineral nutrient accumulation, and essential oil production, while modulating antioxidant activity through improved physiological performance and nutrient availability. Therefore, the present research aimed to evaluate the interactive effects of silicon, nano‐fertilizer, and Disper fertilizer on morpho‐physiological traits, pigment composition, essential oil yield, antioxidant response, and macro‐ and micronutrient contents of basil cultivated under aquaponic conditions with koi fish. Furthermore, this study sought to identify the most efficient fertilizer combination for producing nutritionally enriched basil and to propose a sustainable fertilization approach for high‐quality basil production in water‐limited urban aquaponic systems.

While several research researches are focused on examining the possible advantages and disadvantages of utilizing silicon and nano fertilizers in soil and hydroponic systems, the effects of using both together under aquaponic conditions have not yet been extensively researched. The main factor for the lack of research is that many aquaponic systems have nutritional deficiencies and some aquaponic systems are imbalanced due to nutrient precipitation, competition for nutrients from microbial organisms and limited bioavailability; thus, there is often much less than sufficient quantities of some of the most important nutrients (iron, potassium, zinc, etc.) required to produce healthy and good‐quality crops in aquaponic systems. Therefore, it will be necessary to evaluate the application and the use of silicon and nano fertilizer as well as using chelated micronutrition through foliar spray to impact the growth and nutrient uptake of basil plants, as well as to impact the secondary metabolite production in basil.

## 2 Material and Methods

### 2.1 Growing Conditions and Applying Treatments

The experiment was conducted in the specialized greenhouse of the Department of Horticultural Sciences, University of Mosul, Mosul, Iraq, from September 2024 to September 2025. The greenhouse provided controlled conditions suitable for aquaponic cultivation of basil ( *Ocimum basilicum* L.). The aquaponic units were prepared and their tanks were filled with clean, treated water to remove chlorine in September 2024, nitrogen‐fixing bacteria were activated by providing optimal conditions for their growth (temperature ranged between 20°C and 30°C and pH level maintained at 6.5–7.5). Uniform basil seedlings were obtained from a reliable commercial nursery in Mosul and were rooted in water for 2 months. One month after the system was operated and water was recycled in the aquaponic units, koi fish ( *Cyprinus rubrofuscus* ) were added to system. Then the basil seedlings ( *O. basilicum* ) were transferred to the aquaponic units using expanded clay pellets as the growth substrate. Plants were cultivated under controlled greenhouse conditions throughout the experimental period. The experiment was arranged as a completely randomized design (CRD) with three independent aquaponic systems (biological replicates). Each system contained five plants, resulting in a total of 15 experimental plants. All measurements were conducted at the plant level (*n* = 15), and this definition has been standardized throughout the manuscript.

### 2.2 Aquaponic System Setup

**FIGURE 1:**
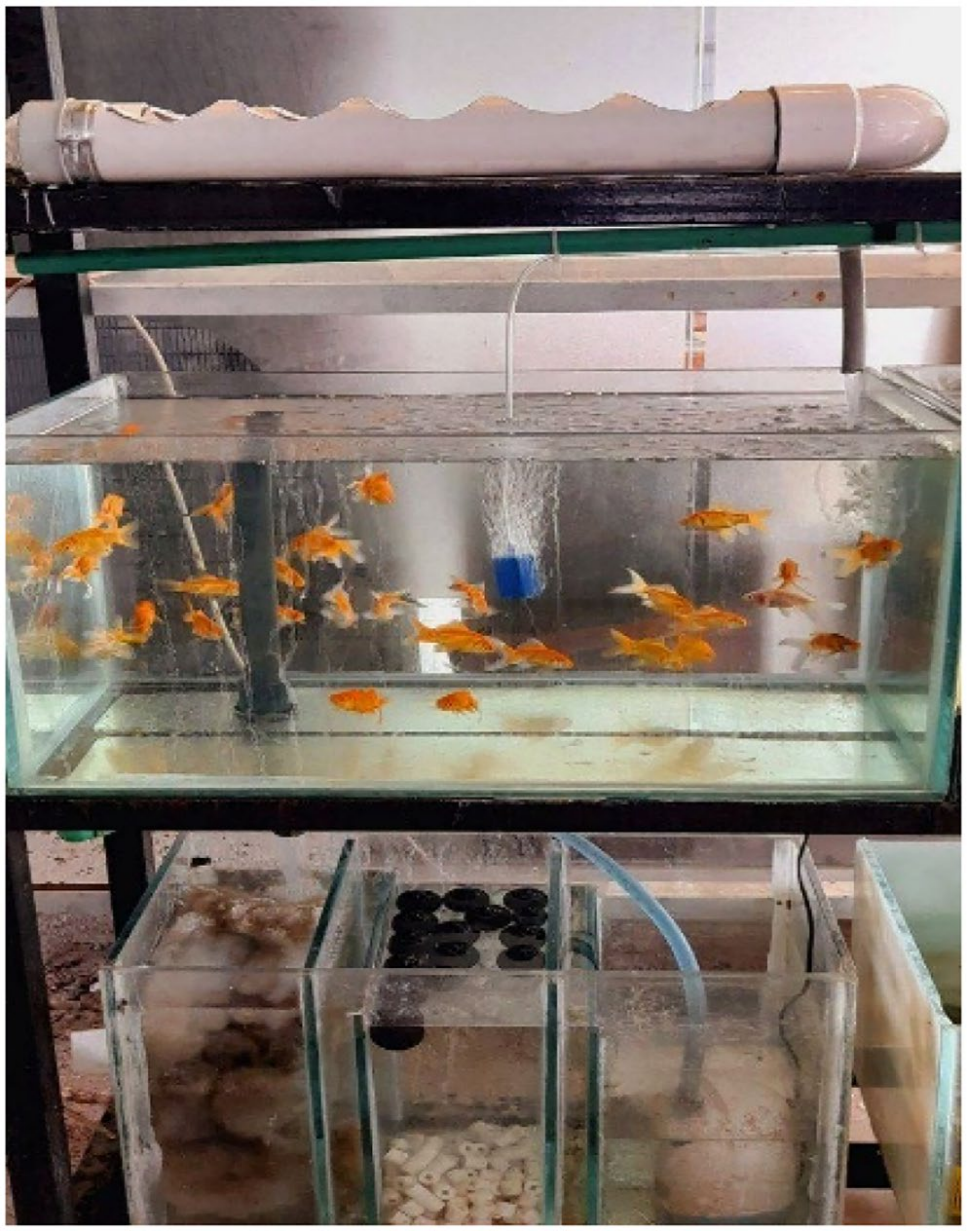
Aquaponic system and components.

The aquaponic units were operated as a closed NFT system within a private nursery greenhouse. Each unit consisted of a multi‐tiered iron frame (150 × 90 cm) with three shelves interconnected by plastic tubing. Upper shelf: Contained NFT channels. Three plastic tubes (60 cm length, 2 in. diameter) were installed, each with five planting holes (7.5 cm diameter) equipped with two anvils (7 cm diameter) for basil seedlings. Middle shelf: Contained a glass fish tank (60 × 30 × 60 cm^3^). Lower shelf: Contained a glass filtration unit (60 × 30 × 60 cm^3^) subdivided into: Mechanical filter: polyethylene mesh for solid waste removal. Biofilter: Bio Balls to support nitrifying bacteria, with a water recirculation pump (Figure 1). Additional components: Air compressor to provide oxygen, water heater to maintain temperature, and LED lights to support plant growth. Operating parameters: Water recirculation rate: 1–2 L min^−1^; pH maintained at 6.5–7.5; temperature ranged between 20°C and 30°C. Nitrogen concentrations (ammonia, nitrate, nitrite) were measured in the central laboratory of the College of Agriculture and Forestry, University of Mosul. Prior to the experiment, the system was biologically cycled to establish nitrifying bacteria, allowing for the conversion of ammonia to nitrate. No external nitrogen compounds were added during the experimental period.

Dissolved oxygen (DO) levels in the aquaponic system were continuously monitored and maintained at 6.5 ± 0.5 mg·L^−1^ using aeration systems, which is within the optimal range for fish and plant performance in aquaponic systems. Light was supplied using full‐spectrum LED panels (model: HLG 550 V2, Horticulture Lighting Group, USA) with an intensity of approximately 250 ± 20 μmol·m^−2^·s^−1^ at canopy level under a 16 h light/8 h dark photoperiod, as recommended for basil cultivation in controlled environments. Fish stocking density was maintained at 20 kg·m^−3^ to ensure sufficient nutrient availability while maintaining system stability. Fish were fed a commercially formulated floating pellet feed containing 32%–38% crude protein at a rate of 2%–3% of body weight per day, divided into two equal meals. Feeding was kept constant across all experimental units throughout the study and was not considered an experimental variable. The feeding regime followed standard aquaponic husbandry practices as reported in previous studies (Rakocy et al. 2006).

### 2.3 Fertilizers and Treatment Levels

The nine combined treatments including Si (0, 0.1, and 0.2 mM) plus a nano‐green fertilizer (Nanogreen) (0, 1, and 2 g L^−1^). Nanogreen fertilizer is a commercial product whose complete nutrient composition was obtained from the manufacturer's technical datasheet (Agrowy Company, Poland). The fertilizer contains 2.4% calcium oxide (CaO) and 0.6% magnesium oxide (MgO), along with N, P, and K macroelements and essential micronutrients as specified by the manufacturer, as well as Disper Complex GS fertilizer at 0, 2, and 4 g L^−1^ (a chelated formula without affecting fish health), manufactured by the Agrowy company, Poland, which contains micronutrients including iron (Fe) 5%, manganese (Mn) 3.5%, zinc (Zn) 0.4%, copper (Cu) 0.4%, and boron (B) 0.6%. Each experimental unit received one of these treatments, and a control group without any supplementation was included. Foliar applications were performed three times during the experimental period, and the aquaponic units were divided according to fertilizer type and concentration to evaluate their effects on basil growth.

Nanogreen fertilizer is a commercial product whose complete nutrient composition was obtained from the manufacturer's technical datasheet (Agrowy Company, Poland). The fertilizer contains 2.4% calcium oxide (CaO) and 0.6% magnesium oxide (MgO), along with N, P, and K macroelements and essential micronutrients as specified by the manufacturer.

### 2.4 Growth and Physiological Measurements

In the end of the experiment plant height (cm), number of branches/plant (n), diameter of basal internodes (mm), fresh weight of shoots (g), and dry weight of roots (g) (after incubation for 48 h in an oven at 70°C) were recorded for all plants in each replication. All chemical analyses were performed according to standard procedures described by AOAC (2019) with minor modifications.

### 2.5 Total Chlorophyll and Carotenoids

Four weeks after foliar application, total chlorophyll concentrations were measured according to Lichtenthaler and Buschmann (2001). Approximately 0.1 g of leaf tissue was finely chopped and ground in a porcelain mortar with 10 mL of 80% acetone. The resulting extract was centrifuged at 3200 rpm for 10 min using a refrigerated centrifuge (R 5810, Eppendorf, Germany). Each extract was then brought to a final volume of 10 mL with 80% acetone, and absorbance was measured using a spectrophotometer (Hitachi U‐1800, Japan) at wavelengths of 663, 646, and 470 nm. 80% acetone without leaf extract was used as a blank (Lichtenthaler and Buschmann 2001). The concentrations of total chlorophyll and carotenoids (mg·g^−1^ fresh leaf weight) were calculated using Equation (1), (2), (3), (4).

(1) $$\text{Chlorophyll}\;a\;\left(\mathrm{mg}\;g^{-1}\;\mathrm{FW}\right)=\left[\left(12.7\times A663\right)-\left(2.69\times A645\right)\right]\times V/\left(W\times 10\right)$$

(2) $$\text{Chlorophyll}\;b\;\left(\mathrm{mg}\;g^{-1}\;\mathrm{FW}\right)=\left[\left(22.9\times A645\right)-\left(4.68\times A663\right)\right]\times V/\left(W\times 10\right)$$

(3) $$\text{Total Chlorophyll}\;\left(\mathrm{mg}\;g^{-1}\;\mathrm{FW}\right)=\left[\left(20.2\times A645\right)+\left(8.02\times A663\right)\right]\times V/\left(W\times 10\right)$$

(4) $$\text{Carotenoids}=\frac{\left[\;\left(1000\times A470\right)-\left(1.82\times \mathrm{Chl}\;a\right)-\left(85.02\times \mathrm{Chl}\;b\right)\right]}{198}$$

### 2.6 Anthocyanins and Antioxidant Activity

The anthocyanins content of leaf was determined based on Wagner (1979). The leaf samples were crushed in acidic methanol solution using a porcelain mortar. Anthocyanin content was determined using acidic methanol extraction, where 0.5 g of fresh leaf tissue was homogenized in 5 mL of methanol containing 1% (v/v) HCl (tissue‐to‐solvent ratio 1:10, w/v), The extract was incubated in darkness at 4°C for 24 h and centrifuged at 10,000 rpm for 10 min, and absorbance was measured at 550 and 657 nm using a spectrophotometer with a 1 cm path length.

Antioxidant activity was estimated with the free radical inhibition (DPPH) method. For antioxidant activity, plant extracts were prepared at a concentration of 1 mg·mL^−1^, and 1 mL of extract was mixed with 2 mL of 0.1 mM DPPH solution (Brand‐Williams et al. 1995). The mixture was incubated in darkness for 30 min, and absorbance was recorded at 517 nm. The DPPH stock solution was prepared by dissolving 0.004 g of DPPH in 100 mL of methanol. A 3.9 mL of a prepared DPPH (2,2‐diphenyl‐1‐picrylhydrazyl) stock solution was added to a test tube. Then, 0.1 mL of each extract was added to the test tube containing the DPPH solution and was reserved in a dark environment for 30 min. The absorbance of the solution was read at 517 nm using an Apel PD‐303 UV spectrophotometer. DPPH radical inhibition percentage was calculated using Equation 5 (Brand‐Williams et al. 1995).

(5) $$I\%=\left[\frac{A_{0}-A_{s}}{A_{0}}\right]\times 100$$

where *I*% represents free radical inhibition percentage, *A*_0_ represents control absorption, and *A*_s_ represents sample absorption.

### 2.7 Essential Oil Extraction

A total of 15 g of dried basil aerial parts were hydrodistilled for 3 h with a Clevenger type apparatus. A fixed water‐to‐plant ratio of 10:1 (v/w) was maintained to ensure consistency in extraction efficiency. The apparatus was calibrated prior to extraction to ensure reproducibility of oil yield. The essential oil obtained from three replications was weighed with a digital scale with an accuracy of 0.0001 g and dehydrated with dry sodium sulfate (Mumivand et al. 2023). The essential oil percentage in each sample was calculated using Equation 6. The yield of essential oil was obtained by multiplying the percentage of essential oil with the dry weight of the aerial parts (g/plant).

(6) EO%=EOweight/Plantdryweight×100

### 2.8 Nutrition Measurement

Leaf nutrients were measured using digested dry and milled samples. Initially, the plant material was washed with distilled water three times. Then, it was dried in an oven at 65°C for 48 h; the dried samples were placed in a furnace and heated at 500°C for 2 h, resulting in the formation of ash. For nutrient analysis, 0.5 g of oven‐dried plant material was digested using a mixture of concentrated HNO_3_ and HCl (3:1, v/v). After digestion, samples were diluted to a final volume of 50 mL with deionized water. Elemental concentrations were determined using ICP‐OES (PerkinElmer Optima 8000). The ash was then dissolved in 4 mL of diluted HNO3 (1:1 ratio) with a density of 1.33. The solution was evaporated using a hot plate at 100°C, leaving behind a residue. The residue was further heated at 500°C for 1 h and allowed to cool. Subsequently, 10 mL of diluted HCl (1:1 ratio) with a density of 1.18 was added to the residue, and heat (100°C) was applied on a hot plate to dissolve the residue. Then total nitrogen concentration (%) was measured using a microkjeldahl nitrogen meter as described by Sáez‐Plaza et al. (2013). Total phosphorus concentration (%) was measured at 420 nm using an Apel PD‐303 UV spectrophotometer (Temminghoff and Houba 2004). Total potassium concentration (%) was estimated using a Shewood 410 flame photometer (Temminghoff and Houba 2004). Finally, iron and zinc micronutrients (mg/kg) were measured using an atomic absorption spectrophotometer (model 240FS, Agilent, Santa Clara, CA, USA) (Sáez‐Plaza et al. 2013).

### 2.9 Data Analysis

The normality test was directed using IBM SPSS software (Ver. 27), then factorial ANOVA and Tukey's HSD test (α = 0.05) were steered after confirming the normality of data via R 4.5.1. Software. Furthermore, Pearson's Correlation Estimation and Cluster Analysis were done using R 4.5.1. Software. As well, Principal component analysis (PCA) and hierarchical cluster analysis (HCA) were performed using were conducted by Minitab software (Ver. 20). Stepwise regression analysis was conducted to determine the relative contribution of mineral nutrients to plant biomass. All measurements were expressed using SI units and spectrophotometric readings were conducted using a 1 cm path length cuvette.

## 3 Results and Discussion

Analysis of variance revealed that treatments had highly significant effects on most growth, physiological, biochemical, and mineral traits (*p* < 0.01), while replication effects were generally non‐significant, indicating minimal experimental variation among replicates. Specifically, plant height, leaf width, plant fresh weight, root dry weight, total chlorophyll, carotenoids, anthocyanin content, antioxidant activity, and essential oil content were all significantly influenced by treatments. Among mineral nutrients, nitrogen showed a modest but significant response to treatment (*p* < 0.05), whereas phosphorus, potassium, iron, and zinc were highly responsive (*p* < 0.01), with zinc exhibiting the largest effect.

### 3.1 Morpho‐Physiological Characteristics

**FIGURE 2:**
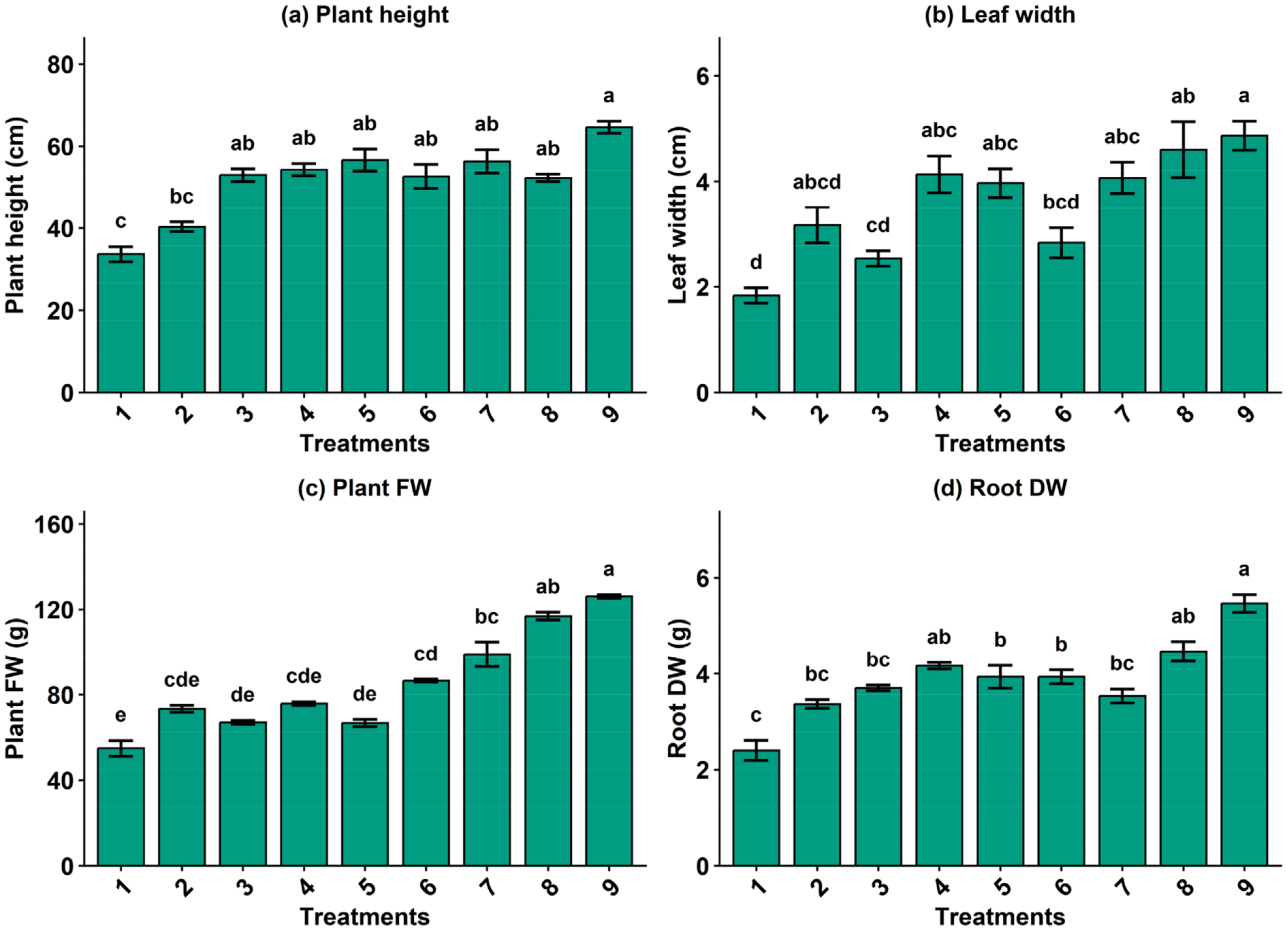
The effect of different combined treatments of Si (mM), nano‐fertilizer (NG; Nanogreen) (g L^−1^), and micronutrient Disper Complex GS fertilizer (g L^−1^) on morpho–physiological attributes of basil plant under integrated aquaponic conditions with koi fish: (a) Plant height, (b) leaf width, (c) plant fresh weight, and (d) root dry weight (Tukey's HSD test, α = 0.05, *n* = 16). T1: Si (0 mM) + NG (0 g L^−1^) + Disper (0 g L^−1^), T2: Si (0.1) + NG (1) + Disper (2), T3: Si (0.1) + NG (1) + Disper (4), T4: Si (0.1) + NG (2) + Disper (2), T5: Si (0.1) + NG (2) + Disper (4), T6: Si (0.2) + NG (1) + Disper (2), T7: Si (0.2) + NG (1) + Disper (4), T8: Si (0.2) + NG (2) + Disper (2), and T9: Si (0.2) + NG (2) + Disper (4).

Plant height was significantly affected by the combined application of silicon, nano green, and Disper (Figure 2a). The lowest plant height was observed in the control treatment (Si 0 mM + NG 0 g L^−1^ + Disper 0 g L^−1^), with a mean value of 33.67 cm, which was statistically inferior to most treated plants. Treatments containing Si at 0.1 and 0.2 mM combined with higher nano green and Disper levels generally produced taller plants. The maximum plant height (64.67 cm) was recorded under Si (0.2 mM) + NG (2 g L^−1^) + Disper (4 g L^−1^), which belonged to the highest statistical group. Overall, under aquaponic system cultivation, silicon‐enriched treatments markedly enhanced basil height compared with the untreated control.

Significant differences in plant fresh weight were detected among treatments (Figure 2b). The control treatment exhibited the lowest fresh weight (54.87 g). Increasing levels of silicon and nano green substantially improved plant biomass. The highest fresh weights were obtained under Si (0.2 mM) + NG (2 g L^−1^) combined with Disper at 2 and 4 g L^−1^, recording 116.93 and 126.13 g, respectively. This enhancement in plant growth may be attributed to improved nutrient uptake efficiency and metabolic activity under the applied treatments, which are known to promote cell division and biomass accumulation.

Leaf width showed a clear positive response to combined treatments. The narrowest leaves were observed in the control plants (1.83 cm) (Figure 2c), which were statistically separated from most other treatments. Application of silicon at 0.1 and 0.2 mM significantly increased leaf width, particularly when combined with higher nano green and Disper concentrations. The widest leaves (4.87 cm) were recorded under Si (0.2 mM) + NG (2 g L^−1^) + Disper (4 g L^−1^). This trend highlights the beneficial role of silicon‐based treatments in improving leaf development.

Root dry weight was also significantly influenced by the treatments. The minimum root DW (2.40 g) was found in the control treatment (Figure 2d). Under NFT aquaponic system cultivation, progressive increases in silicon, nano green, and Disper levels led to a gradual enhancement in root biomass of basil. The highest root dry weight (5.47 g) was obtained with application of Si (0.2 mM) + NG (2 g L^−1^) + Disper (4 g L^−1^), which formed a distinct superior group. These findings demonstrate that combined silicon and nano green application effectively promotes root growth and dry matter accumulation of basil in the aquaponic farming.

### 3.2 Leaf Pigments and Antioxidant Content

**FIGURE 3:**
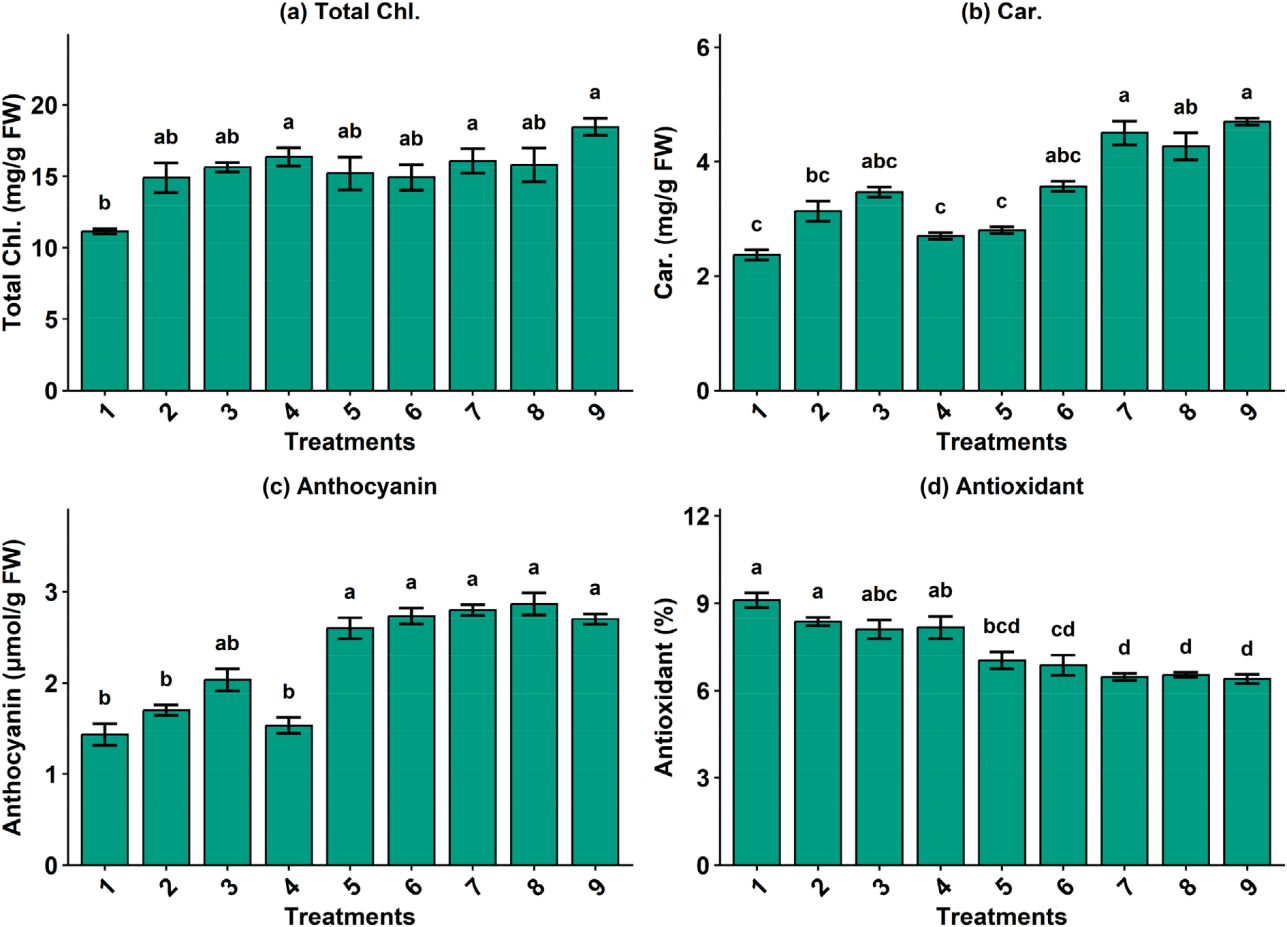
The effect of different combined treatments of Si (mM), nano‐fertilizer (NG; Nanogreen) (g L^−1^), and micronutrient Disper Complex GS fertilizer (g L^−1^) on morpho–physiological attributes of basil plant under integrated aquaponic conditions with koi fish: (a) Total chlorophyll, (b) total carotenoid, (c) anthocyanin content, and (d) antioxidant content (Tukey's HSD test, α = 0.05, *n* = 16). T1: Si (0 mM) + NG (0 g L^−1^) + Disper (0 g L^−1^), T2: Si (0.1) + NG (1) + Disper (2), T3: Si (0.1) + NG (1) + Disper (4), T4: Si (0.1) + NG (2) + Disper (2), T5: Si (0.1) + NG (2) + Disper (4), T6: Si (0.2) + NG (1) + Disper (2), T7: Si (0.2) + NG (1) + Disper (4), T8: Si (0.2) + NG (2) + Disper (2), and T9: Si (0.2) + NG (2) + Disper (4).

Total chlorophyll content of basil leaves was significantly influenced by the combined application of silicon, nano green, and Disper fertilizers under aquaponic planting (Figure 3a). The lowest chlorophyll concentration was observed in the control treatment (11.15 mg g^−1^ FW). In contrast, most treatments receiving silicon at 0.1 and 0.2 mM showed significantly higher chlorophyll levels and were classified in the top statistical groups. The highest chlorophyll content (18.47 mg g^−1^ FW) was recorded under Si (0.2 mM) + NG (2 g L^−1^) + Disper (4 g L^−1^). These results indicate that silicon‐based treatments effectively enhanced photosynthetic pigment accumulation of basil plants in the integrated aquaculture NFT system.

The control treatment produced the lowest carotenoid content (2.37 mg g^−1^ FW), belonging to the lowest statistical group (Figure 3b). Application of silicon combined with nano and micronutrients fertilizers markedly increased carotenoid levels, particularly at higher application rates. The highest carotenoid contents were observed in treatments containing Si (0.2 mM) combined with Nanogreen fertilizer at 1 or 2 g L^−1^ and Disper micronutrient fertilizer at 2 or 4 g L^−1^, with values ranging from 4.27 to 4.70 mg g^−1^ FW, all classified in the highest statistical group. This finding demonstrated a strong stimulatory effect of silicon and nano fertilizer on carotenoid biosynthesis in basil plant under aquaponic system cultivation. The increase in photosynthetic pigments suggests enhanced photosynthetic capacity, likely due to improved nutrient availability and the role of silicon and micronutrients in chloroplast stability and chlorophyll biosynthesis.

Also, anthocyanin accumulation was significantly affected by treatment combinations. Increasing levels of silicon and nano green led to a pronounced increase in anthocyanin concentration (Figure 3c). The lowest anthocyanin content was found in the control treatment (1.43 μmol g^−1^ FW), which clustered in a lower statistical group. However, the highest anthocyanin values (2.70–2.87 μmol g^−1^ FW) were recorded under Si (0.2 mM) combined with nanofertilizer (1–2 g L^−1^) and higher levels of Disper micronutrient fertilizer, all of which were placed in the highest statistical group. These findings suggest enhanced secondary metabolite accumulation in basil plants under aquaponic culture, in response to silicon‐based treatments and nutrient availability.

Furthermore, antioxidant activity also exhibited significant variation across combined treatments; however, an inverse trend was observed compared with pigment accumulation. The highest antioxidant activity was recorded in the control treatment (9.10%), which belonged to the top statistical group. Increasing silicon, nano green, and Disper application resulted in a gradual reduction in antioxidant activity, with the lowest values (6.40%–6.87%) observed under Si (0.2 mM) combined with NG (1–2 g L^−1^) and Disper (2–4 g L^−1^) (Figure 3d). This decline may reflect reduced oxidative stress under improved nutritional conditions in aquaponic culture of basil. The observed variation in antioxidant‐related compounds may reflect changes in plant physiological status, where improved nutrient conditions reduce the need for stress‐induced secondary metabolite production.

### 3.3 Essential Oil and Nutrients Contents

**FIGURE 4:**
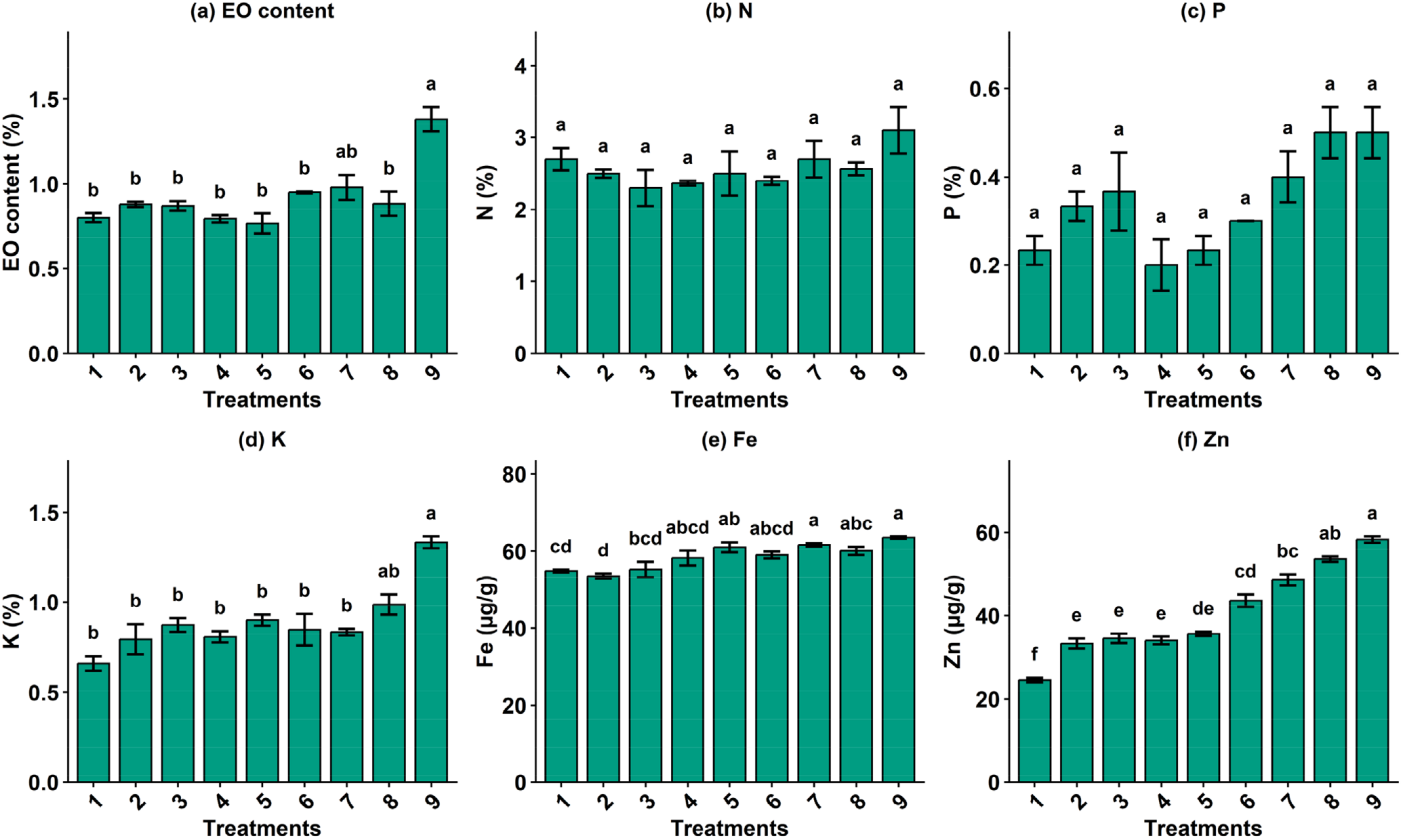
The effect of different combined treatments of Si (mM), nano‐fertilizer (NG; Nanogreen) (g L^−1^), and micronutrient Disper Complex GS fertilizer (g L^−1^) on morpho–physiological attributes of basil plant under integrated aquaponic conditions with koi fish: (a) Essential oil content, (b) nitrogen content of leaves, (c) phosphorus content, (d) potassium content, (e) iron content, and (f) zinc content of leaves (Tukey's HSD test, α = 0.05, *n* = 16). T1: Si (0 mM) + NG (0 g L^−1^) + Disper (0 g L^−1^), T2: Si (0.1) + NG (1) + Disper (2), T3: Si (0.1) + NG (1) + Disper (4), T4: Si (0.1) + NG (2) + Disper (2), T5: Si (0.1) + NG (2) + Disper (4), T6: Si (0.2) + NG (1) + Disper (2), T7: Si (0.2) + NG (1) + Disper (4), T8: Si (0.2) + NG (2) + Disper (2), and T9: Si (0.2) + NG (2) + Disper (4).

Essential oil content was significantly influenced by the combined application of silicon, nano green, and micronutrient fertilizer (Disper). The control treatment recorded a relatively low EO content (0.80%), which was classified in group b. Most treatments receiving silicon at 0.1 and 0.2 mM did not differ significantly from the control and remained within the same statistical group. However, the highest EO content (1.38%) was obtained in response to Si (0.2 mM) + nano fertilizer (2 g L^−1^) + Disper micronutrient fertilizer (4 g L^−1^), which formed a distinct superior group (Figure 4a). This result indicates that higher silicon and nano green levels significantly enhance essential oil accumulation in basil planted under aquaculture conditions.

The effect of fertilizer treatments was not significant for nitrogen content of basil leaves under experimental condition (Figure 4b) with values ranging from 2.3% to 3.1%. The highest nitrogen content (3.1%) was observed under Si (0.2 mM) + NG (2 g L^−1^) + Disper (4 g L^−1^); however, this increase was not statistically significant compared with other treatments. The phosphorus content varied significantly across treatments. The lowest P concentrations were observed in the control (0.23%) and some treatments with lower nano green and Disper levels. In contrast, treatments receiving higher silicon and nano green levels showed increased phosphorus accumulation. The maximum P content (0.50%) was recorded under Si (0.2 mM) + nanogreen fertilizer (2 g L^−1^) combined with micronutrient fertilizer (Disper at 2 or 4 g L^−1^), both of which were classified in the highest statistical group (Figure 4c). These evidences demonstrated a positive role of silicon‐based treatments in enhancing phosphorus uptake with basil plant under aquaponic cultivation. Also, the effect of different treatments was significant on potassium content. The control treatment exhibited the lowest potassium concentration (0.66%), forming a distinct inferior group (Figure 4d). Application of silicon combined with nano green and Disper significantly increased potassium content. The highest K value (1.33%) was obtained under Si (0.2 mM) + NG (2 g L^−1^) + Disper (4 g L^−1^). Overall, nutrients‐enriched treatments markedly improved potassium accumulation compared with untreated basil plants. Iron concentration responded significantly to treatment combinations. The control plants showed relatively low Fe content (54.87 μg g^−1^). Increasing silicon and nano green application generally enhanced iron accumulation (Figure 4e). The highest iron content (63.6 μg g^−1^) was recorded under Si (0.2 mM) + NG (2 g L^−1^) + Disper (4 g L^−1^) (T9), which formed the top statistical group (a). These results indicate that silicon and nano green application can effectively improve iron uptake. Finally, Zinc content was strongly affected by the applied treatments. The control treatment recorded the lowest Zn concentration (24.6 μg g^−1^), belonging to the lowest statistical group (Figure 4f). A gradual and significant increase in zinc content was observed with increasing silicon, nano green, and Disper levels. The maximum Zn concentration (58.3 μg g^−1^) was obtained under Si (0.2 mM) + NG (2 g L^−1^) + Disper (4 g L^−1^) (T9), which was classified in the highest statistical group. This highlights the strong synergistic effect of silicon and nano green on zinc accumulation in basil plants under aquaponic farming system. Differences in nutrient accumulation among treatments may be associated with enhanced root activity and nutrient transport mechanisms influenced by silicon and nano‐fertilizer applications.

### 3.4 Results of Pearson's Correlation

**FIGURE 5:**
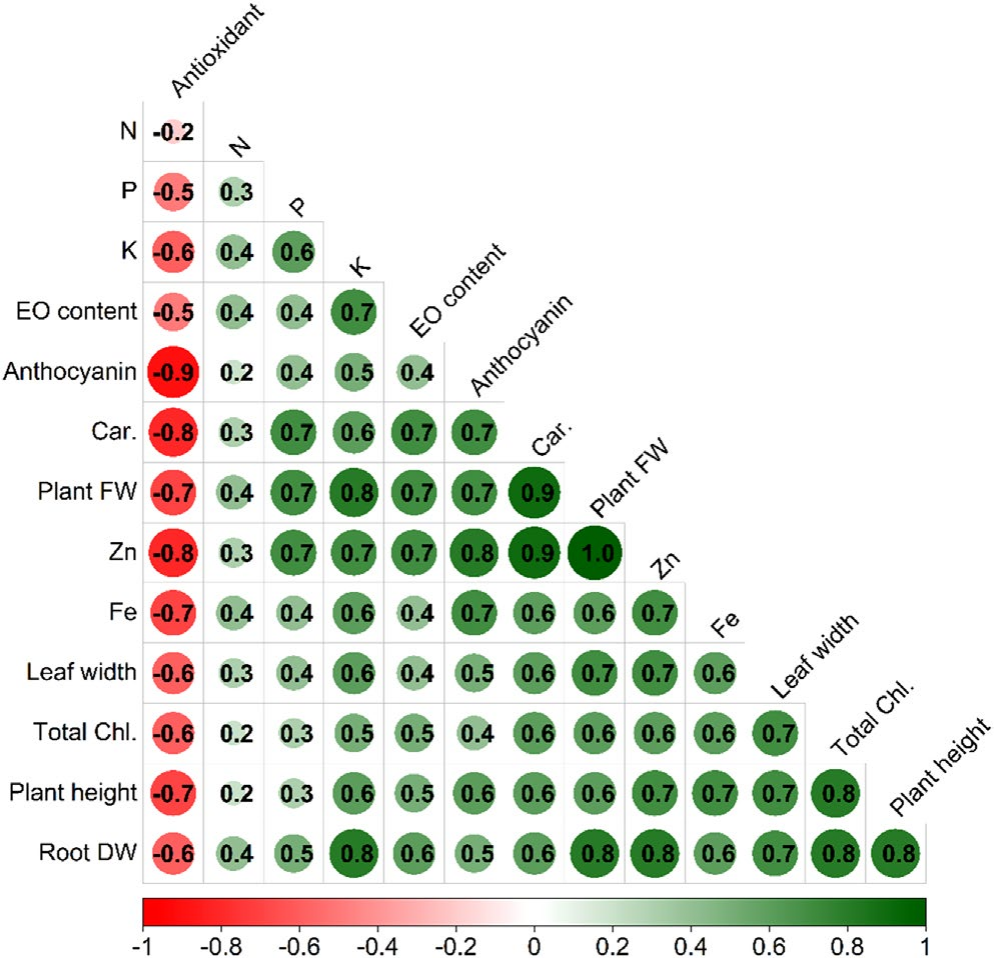
The correlation matrix for different studied traits of basil plant under integrated aquaponic conditions with koi fish and effects of fertilization with combined treatments of Si (mM), nano‐fertilizer (NG; Nanogreen) (g L^−1^), and micronutrient Disper Complex GS fertilizer (g L^−1^).

Correlation analysis (Figure 5) revealed a complex and highly coordinated network of positive associations among morphological, physiological, biochemical, and mineral traits, indicating that plant growth, pigment accumulation, and nutrient uptake are tightly interconnected. Plant height and leaf width were strongly correlated with biomass production, photosynthetic pigments, and mineral nutrients, demonstrating that canopy development is closely linked to enhanced physiological performance and nutrient status. Plant fresh weight showed particularly strong positive associations with root dry weight, carotenoids, anthocyanin, essential oil content, and the macronutrients phosphorus and potassium, as well as with the micronutrients iron and zinc, confirming that both root development and mineral nutrition are major determinants of biomass accumulation. The exceptionally strong correlation between plant fresh weight and zinc further supports the regression results identifying Zn as a key driver of growth. Photosynthetic pigments were also highly interrelated and positively associated with root biomass and micronutrient content, suggesting that improved nutrient acquisition promotes chlorophyll synthesis and secondary metabolite production. In contrast, antioxidant activity exhibited consistent and strong negative correlations with most growth, pigment, and mineral traits, indicating that plants with enhanced nutritional status and vigorous growth experienced reduced oxidative stress. Essential oil content was positively linked to biomass, carotenoids, potassium, and zinc, highlighting the importance of mineral nutrition in regulating secondary metabolite biosynthesis. Collectively, these relationships demonstrate that improved mineral uptake, particularly of potassium, phosphorus, and zinc, promotes coordinated increases in root development, photosynthetic capacity, biomass production, and secondary metabolism, while simultaneously alleviating oxidative stress, underscoring the central role of nutrient‐mediated physiological integration in determining overall plant performance.

### 3.5 Principal Component Analysis (PCA)

Principal component analysis revealed that the first two principal components (PC1 and PC2) explained 72% of the total variance, with PC1 accounting for 63% and PC2 for 9% of the variability. PC1 was strongly and positively associated with most growth, physiological, and nutritional traits (Table 1). In contrast, antioxidant activity showed a strong negative loading on PC1, indicating an inverse relationship with plant growth and nutrient accumulation. Therefore, PC1 can be interpreted as a growth, nutrient uptake, and productivity axis. PC2 was mainly influenced by phosphorus and nitrogen, with strong negative loadings, while plant height and total chlorophyll content showed notable positive contributions (Table 1). This component reflects a physiological–nutrient balance axis, separating treatments based on pigment accumulation versus macronutrient concentration. Overall, the PCA highlighted strong positive interrelationships among growth traits, mineral nutrients, and photosynthetic pigments, while antioxidant activity was negatively associated with these parameters.

**TABLE 1:** The loading share of different variables in the 1 and 2 PCs for basil plants under aquaponic cultivation.

Variables	PC1	PC2	Variables	PC1	PC2
Plant height	0.273	0.438	Antioxidant	−0.29	−0.05
Leaf width	0.259	0.264	EO content	0.245	−0.189
Plant FW	0.312	−0.191	N	0.139	−0.37
Root DW	0.292	0.176	P	0.222	−0.506
Total Chl.	0.248	0.369	K	0.277	−0.095
Carotenoid	0.292	−0.226	Fe	0.262	0.181
Anthocyanin	0.26	0.009	Zn	0.324	−0.102
Eg. value	0.245	−0.189	Eg. value	8.84	1.22
V (%)	0.139	−0.37	V (%)	0.63	0.09
Cumulative V (%)	0.222	−0.506	Cumulative V (%)	0.63	0.72

**FIGURE 6:**
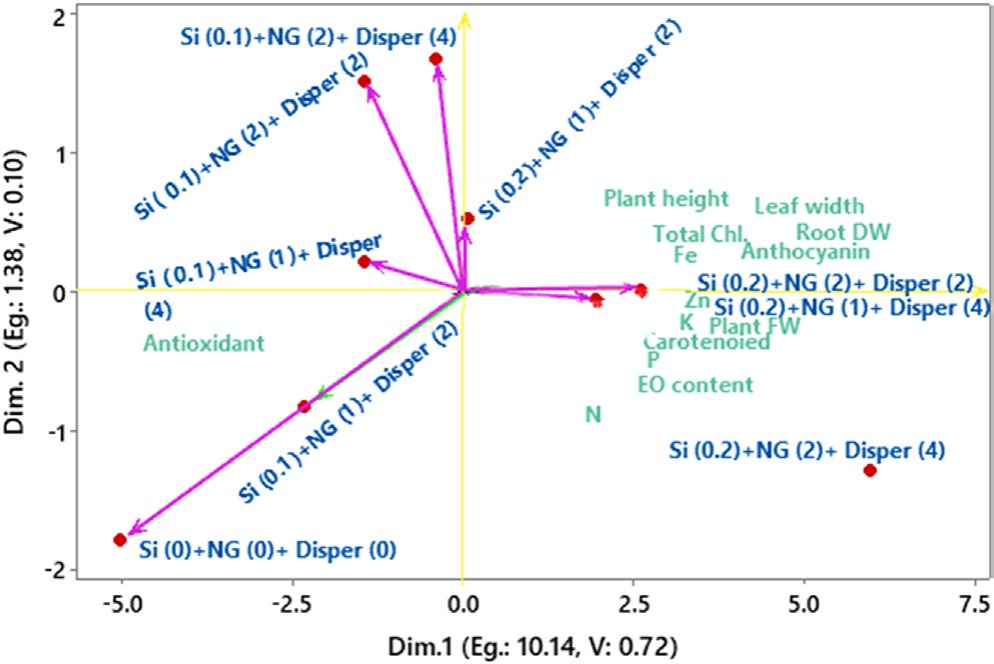
The PCA graph of different studied traits and combined treatments of Si (mM), nano‐fertilizer (NG; Nanogreen) (g L^−1^), and micronutrient Disper Complex GS fertilizer (g L^−1^) for basil plant under integrated aquaponic conditions with koi fish.

The PCA graph (Figure 6) exhibits the relationships between treatments and measured traits based on the first two principal components, which together explained 72% of the total variance (PC1 = 63%, PC2 = 9%). The horizontal axis (PC1) clearly separated treatments according to overall plant growth, biomass production, pigment content, essential oil accumulation, and mineral nutrient uptake. The treatments of Si (0.2 mM) + NG (2 g L^−1^) + Disper (4 g L^−1^) and Si (0.2 mM) + NG (1 g L^−1^) + Disper (4 g L^−1^), were strongly associated with plant height, leaf width, root dry weight, plant fresh weight, total chlorophyll, carotenoids, anthocyanin, essential oil content, K, Fe, and Zn, indicating superior growth and physiological performance under higher silicon and nano green application rates. In contrast, the control treatment Si (0 mM) + NG (0 g L^−1^) + Disper (0 g L^−1^) closely aligned with antioxidant activity, reflecting poorer growth performance and elevated oxidative stress in untreated basil plants under aquaponic conditions. This inverse positioning confirms the strong negative relationship between antioxidant activity and most growth‐related traits observed in the correlation analysis. The vertical axis (PC2) primarily distinguished treatments based on chlorophyll content and plant height (positive PC2) versus nitrogen and phosphorus content (negative PC2). Treatments such as Si (0.1 mM) + NG (2 g L^−1^) + Disper (4 g L^−1^) and Si (0.1 mM) + NG (2 g L^−1^) + Disper (2 g L^−1^) were positioned higher along PC2, indicating a stronger association with photosynthetic pigments and shoot development. Conversely, treatments located in the lower quadrants showed a greater association with nitrogen content. Overall, the PCA biplot demonstrated that increasing silicon concentration in combination with nano green and Disper fertilizer shifts treatments toward improved growth, enhanced photosynthetic capacity, higher essential oil accumulation, and greater micronutrient uptake, while untreated or low‐input treatments were characterized by higher antioxidant activity and reduced physiological performance. The separation of treatments along PC1 indicates a gradient from low‐performance to high‐performance conditions, primarily driven by differences in biomass and nutrient accumulation.

### 3.6 Cluster Analysis

**FIGURE 7:**
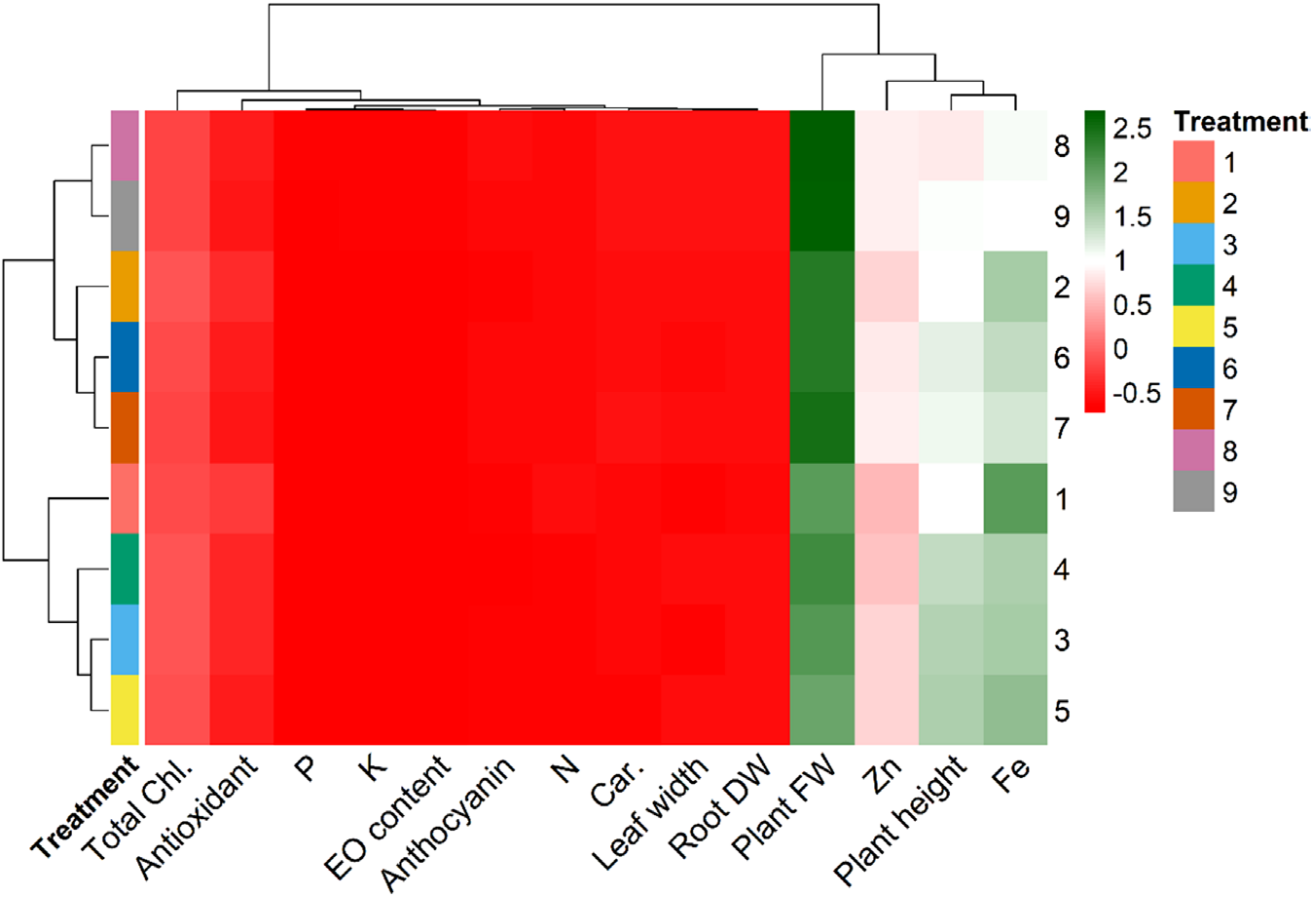
The cluster heatmap for different treatments and studied traits of basil plant under integrated aquaponic conditions with koi fish and effects of fertilization with combined treatments of Si (mM), nano‐fertilizer (NG; Nanogreen) (g L^−1^), and micronutrient Disper Complex GS fertilizer (g L^−1^). T1: Si (0 mM) + NG (0 g L^−1^) + Disper (0 g L^−1^), T2: Si (0.1) + NG (1) + Disper (2), T3: Si (0.1) + NG (1) + Disper (4), T4: Si (0.1) + NG (2) + Disper (2), T5: Si (0.1) + NG (2) + Disper (4), T6: Si (0.2) + NG (1) + Disper (2), T7: Si (0.2) + NG (1) + Disper (4), T8: Si (0.2) + NG (2) + Disper (2), and T9: Si (0.2) + NG (2) + Disper (4).

Cluster analyzing distinctly classified the treatments into three groups (Figure 7). A subcluster included the treatments of 1, 3, 4, and 5 with lower biomass production and lower contents of Fe and Zn. While it confirmed the results of turkeys test and PCA, where demonstrated the combination of Si (0.2 mM) + nano fertilizer (2 g L^−1^) + Disper (4 g L^−1^) (T9) and Si (0.2 mM) + nano fertilizer (2 g L^−1^) + Disper (2 g L^−1^) (T8) as the most superior fertilizer combinations for basil production under the aquaponic system. hierarchical cluster analysis grouped the treatments into three distinct clusters based on biomass production and micronutrient accumulation. The first cluster consisted of low‐performing treatments characterized by reduced growth and nutrient content, whereas the second cluster represented intermediate responses. Notably, T8 and T9 formed a distinct high‐performance cluster, indicating their superior effect on plant growth and nutrient enrichment. The relatively large distance separating this cluster from others further highlights the strong treatment effect.

### 3.7 Results of Regression

**FIGURE 8:**
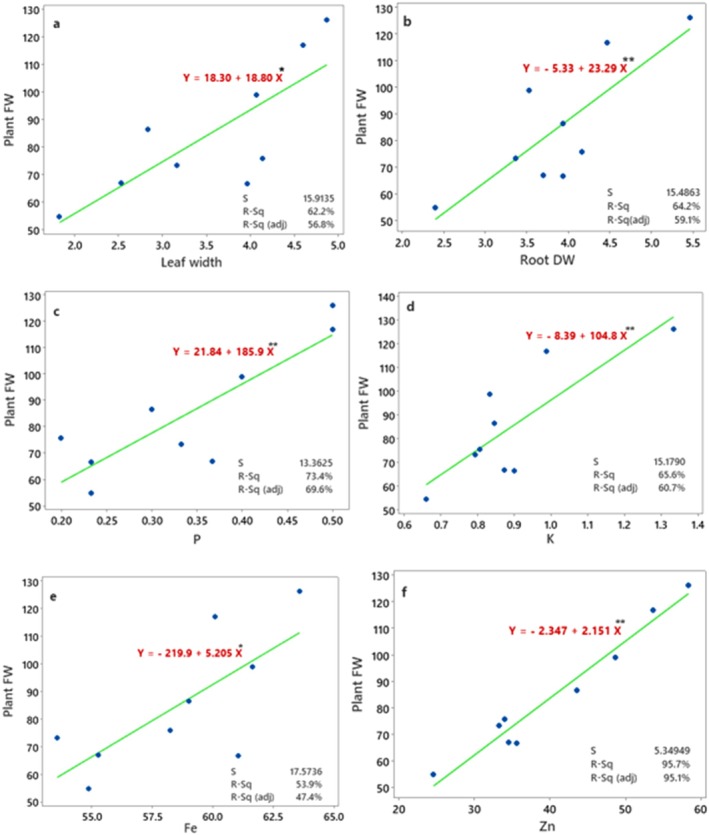
The regression diagram between plant yield as response and significant predictors including leaf width (a), root dry weight (b), phosphorus content (c), potassium content (d), iron content (e), and zinc content (f) of basil leaves under integrated aquaponic conditions with koi fish and effects of fertilization with combined treatments of Si, nano‐fertilizer (NG), and micronutrient Disper Complex fertilizer.

Linear regression analysis between plant fresh mass (dependent variable) and leaf width (predictor) revealed a strong statistically significant and positive association between the predictor and response variable, with the model explaining 62.23% of the total variance (*R* ^2^ = 0.622) (Figure 8a). The regression equation was *Y* = 18.30 + 18.80 *X*. The adjusted coefficient of determination (*R* ^2^ adj = 0.568) remained high, indicating that the model retained substantial explanatory power after accounting for model complexity. The standard error of the regression (*S* = 15.91) reflects moderate dispersion of the residuals around the fitted line. Together, the linear model provides a robust and reliable description of the relationship between the variables. Linear regression analysis demonstrated a significant positive relationship between root dry weight (predictor) and plant fresh weight (*Y* = −5.33 + 23.29 *X*) (Figure 8b). The model was statistically significant (*F* = 12.57, *p* = 0.009) and explained 64.23% of the total variance in plant fresh weight (*R* ^2^ = 0.642; adjusted *R* ^2^ = 0.591), indicating strong explanatory power. The positive slope indicates that increases in root dry weight were associated with substantial increases in plant fresh weight. The standard error of the estimate (*S* = 15.49) suggests moderate dispersion of residuals around the fitted regression line. Together, these results indicate that root biomass is a strong predictor of whole‐plant fresh mass. The differential contribution of mineral nutrients to biomass accumulation may be attributed to their distinct roles in plant metabolism. Micronutrients are essential for enzyme activation and photosynthetic processes, while macronutrients contribute to structural development and energy transfer, which collectively influence plant growth performance. The linear regression between phosphorus concentration and plant fresh weight (*Y* = 21.84 + 185.9 *X*) exhibited a strong and statistically significant positive relationship (Figure 8c). The model was highly significant (*F* = 19.29, *p* = 0.003) and accounted for 73.37% of the total variation in plant fresh weight (*R* ^2^ = 0.734; adjusted *R* ^2^ = 0.696), indicating a high degree of explanatory power. The large positive slope indicates that increases in phosphorus content were associated with pronounced increases in plant fresh biomass. The standard error of the estimate (*S* = 13.36) reflects relatively low residual variability around the fitted regression line. These results demonstrate that phosphorus is a strong predictor of plant fresh weight in this plant.

Also, linear regression analysis (Figure 8d) indicated a significant positive relationship between potassium content (predictor) and plant fresh weight (*Y* = −8.39 + 104.8 *X*). The regression model was statistically significant (*F* = 13.37, *p* = 0.008) and explained 65.64% of the variance in plant fresh weight (*R* ^2^ = 0.656; adjusted *R* ^2^ = 0.607), indicating strong clarifying power. The positive regression coefficient shows that increases in potassium content were associated with substantial increases in plant fresh biomass. The standard error of the estimate (*S* = 15.18) suggests moderate dispersion of residuals around the fitted regression line. These results demonstrate that potassium is a significant predictor of plant fresh weight in basil plants.

The relationship between iron concentration as predictor and plant fresh weight as response variable (*Y* = −219.9 + 5.205 *X*) was statistically significant (*p* = 0.024) and positive (Figure 8e). The model explained 53.94% of the total variation in plant fresh weight (*R* ^2^ = 0.539; adjusted *R* ^2^ = 0.474), indicating a moderate level of descriptive power, where increasing iron content was associated with increased plant fresh biomass. The standard error of the estimate (*S* = 17.57) reflects moderate residual variability around the fitted regression line.

Finally, an exceptionally strong and highly significant positive relationship (Figure 8f) was observed between zinc concentration (independent variable) and plant fresh weight (*Y* = −2.347 + 2.151 *X*). The model was highly significant (*p* < 0.001) and explained 95.73% of the total variance in plant fresh weight (*R* ^2^ = 0.957; adjusted *R* ^2^ = 0.951), indicating an excellent fit and very high predictive power. The steep positive slope demonstrates that increases in zinc concentration were closely associated with increases in plant fresh biomass. The low standard error of the estimate (*S* = 5.35) reflects minimal residual dispersion around the fitted regression line. These results identify zinc as the strongest predictor of basil growth among the variables examined.

Overall, plant fresh weight exhibited significant positive linear relationships with root dry weight and with the concentrations of several macro‐ and micronutrients, indicating that both biomass allocation and mineral nutrition strongly influence plant growth. Root dry weight explained 64.2% of the variation in plant fresh weight, confirming that increased root biomass is closely associated with enhanced shoot growth and whole‐plant mass. Among the macronutrients, phosphorus showed the strongest association with plant fresh weight, followed by potassium, highlighting the central roles of P and K in biomass accumulation and metabolic activity. Iron also exhibited a significant, though weaker, relationship with plant fresh weight, suggesting that micronutrient availability contributes to growth but explains less of the observed variability than the major nutrients. Notably, zinc displayed an exceptionally strong positive relationship with plant fresh weight, accounting for 95.7% of the total variance, identifying Zn as the most powerful predictor among the variables examined. Collectively, these results demonstrate that plant fresh biomass is tightly regulated by both root development and nutrient status, with particularly strong dependence on phosphorus and zinc availability, underscoring the integrated roles of nutrient acquisition and allocation in determining plant growth performance. The positive relationships between growth parameters and nutrient concentrations indicate that improved nutrient availability directly supports metabolic processes and biomass production.

## 4 Discussion

The findings of the present study demonstrated that the application of combined silicon, Disper fertilizer, and Nanogreen, especially a combination of 0.2 mM Si + 2 g L^−1^ nano fertilizer + 4 g L^−1^ Disper, promotes plant growth and dry matter accumulation, phytopigments, EO content, and mineral concentration in basil leaves under aquaponic farming. In an aquaponics study, the basil plants exhibited high growth performance and basil leaf area, fresh herbage yield, and root weight were increased by up to 27%, 11%, and 11%, respectively compared to soil culture (Albadwawi et al. 2022). Furthermore, biomass and height improvements were reported in basil plants grown in decoupled aquaponics (Rodgers et al. 2022). In line with our results, in tomato aquaponic, the 250 ppm Zn‐MPs treatment significantly increased plant yield (+74.9%) and fruit yield (+44.4%) compared to control (Frassine et al. 2025). The regression and correlation analyses further support these findings, indicating that nutrient availability plays a central role in determining biomass accumulation. The observed relationships suggest that improvements in nutrient uptake directly enhance metabolic activity and plant growth.

### 4.1 Physiological and Antioxidant Responses of Basil to Treatments

In current study the content of photosynthetic pigments and anthocyanin significantly improved under supplementation of combined fertilizers. Many reports confirmed that under aquaponic plantation the leave necrosis was occurred and concentration of chlorophyll were decreased (Rupasinghe and Kennedy 2010). There was visible chlorosis, as reported by Roosta (2014), in basil plants cultivated under different aquaponic solutions. Leaf chlorosis can be considered as an indicator of less physiological functions under aquaponics (Mullis and Reyes 2019; Yang and Kim 2019). However, according to some study, when basil is aquaponically cultivated, the SPAD values in the leaves remain unchanged compared to hydroponic and soil culture (Rodgers et al. 2022). The substantial increase observed in all the photosynthetic pigments (both chlorophyll and carotenoid) as a result of the combined treatments indicates that enhanced photosynthetic capacity and metabolic activity have occurred. The increased chlorophyll content is frequently associated with increased nitrogen uptake and a better nutritional condition. Carotenoids play a protective role in preventing oxidative damage to the photosystems. Silicon's positive effects on the structure and function of the chloroplast have also been documented, which has led to improved photosynthetic performance. The eco‐fertilizer suggests there has been a strengthening of secondary metabolism. Secondary metabolites such as essential oils in basil are also related to the plant's nutritional condition and the surrounding environment. The improved availability of micronutrients particularly zinc and iron has likely stimulated the activity of enzymes that are involved in producing secondary metabolites. Similar results have been found in many medicinal and aromatic plants; providing improved nutrients resulted in an increase in biogenic (i.e., bioactive) compounds. The optimization of nutrients in aquaponic, through nano‐fertilization, allows nutrients enter to the plant cell by epidermis and allow to gradual release, targeted distribution, and reducing nutrients excess, as well as enhance nutrient use efficiency (Ain et al. 2023). Thus, in our study, adequate nutrients availability, especially Si, Fe and Zn, a key driver of growth and Photosynthetic pigments maybe helps basil plants for pigments biosynthesis and nonappearance of leaf necrosis, as well as growth improvement, which previously confirmed by Shoukat et al. (2025). Additionally, anthocyanin content in aquaponic strawberries plants has improved compared to control fruits, which is in line with present results (Korbee et al. 2025).

We observed that antioxidant activity was lower in the fertilizer‐treated basil plants compared to control. Usually, the plants under aquaponic growth are probably subjected to nutrient toxicity and a type of water stress (flooding), which in turn increased the antioxidant content in the plants (Rodgers et al. 2022). In the *Acmella oleracea* (L.) the content of total phenolics, flavonoids, and antioxidants in plants cultivated under hydroponic condition was higher than in field and tissue culture (Abeysinghe et al. 2014). It can be concluded that in our study, the application of nanomaterials and silicon reduced the adverse effects of nutrient toxicity and stress in basil plants as previously stated by many authors (Abou El‐Nasr et al. 2025; González‐García et al. 2021; Tighe‐Neira et al. 2023). The inverse relationship between antioxidant activity and growth parameters may be explained by resource allocation theory, where plants prioritize primary metabolism and biomass production under optimal nutrient conditions, resulting in reduced synthesis of stress‐related secondary metabolites.

### 4.2 Nutritional and Mineral Accumulation

The application of Si integrated with nano nutrients significantly enhanced the content of microelements and macroelements in basil leaves. Aquaponics culture, with recirculating water, contains more nutrients, and this helps the basil plant to grow faster (Albadwawi et al. 2022). The waste generated by fish in the aquaponics system tends to deliver essential nutrients for plant growth, such as nitrogen, calcium, magnesium, and potassium (Rakocy et al. 2006). A recent report confirmed that, in an aquaponic system, the contents of nitrogen, boron, and phosphorus were higher than that of the soil system, while the content of potassium, zinc, and calcium exhibited a lower content (Albadwawi et al. 2022). Furthermore, the concentrations of micronutrients Fe, Zn, and B were significantly higher in spinach grown in the aquaponics than in hydroponics, whereas concentrations of Cu and Mn were similar in both systems (Atique et al. 2022). Microbial activity within the aquaponic system likely enhanced nutrient mineralization and availability, thereby increasing the uptake of macro‐ and micronutrients in basil leaves and contributing to improved nutritional quality. In hydroponic systems, silicon (Si) supplementation has been shown to enhance plant growth, photosynthetic efficiency, and stress tolerance, with studies reporting that foliar or nutrient‐solution Si application improves photosystem efficiency and increases biomass in hydroponically grown crops (Shoukat et al. 2024). Si also promotes root development, chlorophyll content, water use efficiency, and antioxidant responses in hydroponic lettuce, demonstrating multiple physiological benefits of Si under soilless cultivation. The beneficial effects of silicon may be attributed to its role in reinforcing cell wall structure, improving mechanical strength, and enhancing stress tolerance. Additionally, micronutrients such as iron and zinc are essential for enzymatic activity and chlorophyll biosynthesis, contributing to improved photosynthetic performance and plant growth. Significant improvements were seen with respect to the accumulation of both macro and micronutrients (especially phosphorus, potassium, iron, and zinc) as a result of this study. The results obtained can be attributed to the combined influence of increased root functioning established as a result of silicon, enhanced nutrient delivery of the nano‐fertilizer system, and the high bioavailability of the chelated micronutrients. In aquaponics, nutrient availability is also restricted due to precipitation reactions and microbial competition. By applying nutrients to the leaves, a new and efficient alternative pathway for nutrient uptake is attained and the limitations to root absorption associated with recirculating systems are avoided.

Microorganisms in aquaponic systems are essential for transforming nutrients and maintaining water quality. They contribute to these processes through various pathways, including organic matter decomposition, mineralization, nitrification, and denitrification. Organic matter decomposition and mineralization break down residual feed and fish excreta into macro‐ and micronutrients, making them available for microbial and plant growth (Goddek, Espinal, et al. 2016). The proportion of feed that is converted into fish biomass and metabolism varies depending on the aquaculture setup, fish species, and feed (Korbee et al. 2025). Minerals such as calcium and magnesium, as well as trace elements, such as iron, zinc, copper, manganese, and molybdenum, are only present in insoluble forms within residual feed or feces (Goddek, Schmautz, et al. 2016). Thus, the type and composition of feed impact microbial communities by influencing nutrient availability and waste breakdown in the system (Korbee et al. 2025). Heterotrophic bacteria contribute to decomposition and mineralization by breaking down organic matter and releasing essential ions for plant assimilation (Delaide et al. 2017; Kasozi et al. 2021). However, plants may experience deficiencies in certain micronutrients, including calcium, iron, magnesium, sodium, and silicon, due to slow and incomplete nutrient remineralization, leading to locked nutrients (i.e., nutrients bound in insoluble compounds or organic complexes) (Lobanov et al. 2021). In present study, application of Si and nano form of micro and macro elements optimized uptake and nutrients availability in the plant cell, resulting enhanced nutrient use efficiency (Ain et al. 2023). It be noted that, due to toxic effect of high ammonia concentrations (above 1 mg/L) to plant and fish, aquaponic systems, nitrifying microbes convert ammonia into nitrate through nitrification (Joo et al. 2005; Korbee et al. 2025).

## 5 Conclusion

The findings of this study indicate that combining silicon (Si), nano‐fertilizers, and chelated micronutrients has a synergistic relationship in an aquaponics setting, influencing growth and physiological characteristics, nutrient uptake, and producing essential oils from plants ( *Ocimum basilicum* ) grown with these treatments. Hydroponically growing basil with 0.2 mM Si, 2 g L^−1^ nano‐fertilizer, and 4 g L^−1^ Disper produced the best results based on the parameters measured. These positive changes were likely due to increased nutrient availability and uptake, as well as improved photosynthesis and greater crop development overall. Additionally, the decreased antioxidant activity suggests a lower rate of oxidative stress related to nutrients supplied. In conclusion, it appears that using silicon‐based integrated nutrient management strategies is both an effective and sustainable method of producing basil in aquaponic systems. Further research should focus on looking at these treatments in combination and analyzing their effects over time to determine their long‐term impact on system stability.

## Author Contributions

**Al‐Hatem JihanYahya:** conceptualization, project administration, methodology. **AsmaaMohammed Adil:** investigation, visualization, formal analysis, data curation. **AliFarouq Al‐Ma'athedi:** validation, project administration, visualization, writing – original draft. **Heidar Meftahizade:** writing – review and editing, software, data curation.

## Funding

The authors have nothing to report.

## Ethics Statement

The authors have nothing to report.

## Consent

The authors have nothing to report.

## Conflicts of Interest

The authors declare no conflicts of interest.

## Data Availability

The data that support the findings of this study are available from the corresponding author upon reasonable request.
